# Integration of One Health activities into professional student education: successes, challenges, and considerations

**DOI:** 10.3389/fmed.2026.1756078

**Published:** 2026-02-16

**Authors:** Karen Gruszynski, MaryBeth Babos, Reagan Bishop, Debra Sullivan, LaRoy Brandt

**Affiliations:** 1 College of Graduate Studies, Midwestern University, Downers Grove, IL, United States; 2 College of Health and Public Administration, Franklin University, Columbus, OH, United States; 3 College of Osteopathic Medicine, Midwestern University, Downers Grove, IL, United States; 4 College of Health Sciences, Clinical Skills and Simulation Center, Midwestern University, Glendale, AZ, United States; 5 Department of Biology and Chemistry, College of Mathematics, Sciences and Health Professions, Lincoln Memorial University, Harrogate, TN, United States; 6 College of Mathematics, Sciences and Health Professions, Cumberland Mountain Research Center, Lincoln Memorial University, Harrogate, TN, United States

**Keywords:** collaborative practice, faculty role modeling, health professions education, interdisciplinary, Interprofessional Education, IPE, One Health

## Abstract

Interprofessional Education (IPE) creates opportunities for multiple disciplines to learn from each other and develop soft skills to improve patient care. One Health similarly emphasizes collaborative and interdisciplinary practice while recognizing the interconnectedness between human, animal, and environmental health. Despite the intersection of IPE and One Health, the meaningful integration of One Health within IPE remains uneven and difficult to operationalize. This perspective synthesizes experiences from multi-college programs implementing One Health-oriented IPE, highlighting successes, challenges, and structural requirements. We argue that for One Health to be successfully integrated into IPE for professional students, it requires extensive logistical planning, coordinated institutional support, modeling of cross-disciplinary collaboration by faculty, and must extend beyond global-scale concepts to incorporate applications to future clinical practice.

## Introduction

Interprofessional Education (IPE) is defined as “occasions where learners of two or more professions learn with, from, and about each other to improve collaboration and the quality of care and services” (1, 2). IPE has been increasingly acknowledged for its critical role in equipping students to collaborate across various healthcare sectors. Engaging with other health professions enhances students’ appreciation of the value of different professions, as well as their own, and highlights the benefits of multiple perspectives (3). Beyond fostering an appreciation for diverse perspectives, IPE has achieved significant adoption, with the Association of American Medical Colleges reporting that 97% of human health IPE programs (involving doctors, nurses, pharmacists, and technicians) have implemented such initiatives (1). While often focused on direct patient care, IPE plays a critical role in developing broader essential healthcare skills, such as leadership, communication, medication plans/errors, and interprofessional knowledge, attitudes, and skills (3, 4). These activities provide opportunities for students to practice these skills before their clinical rotations (5).

Interprofessional Education competencies as well as One Health encompass broad areas, including teamwork, communication, collaboration, ethics, and problem-solving, which is advantageous in allowing flexibility in developing individual priorities and programs (1, 2, 6). Human health professionals are more likely to gravitate toward the term “IPE,” whereas veterinary health professionals embrace the term “One Health” when it comes to interdisciplinary teamwork (1). Where IPE and One Health diverge is that One Health aims to sustainably balance and optimize the health of people, animals, and ecosystems (7). One Health recognizes the interdependence of human, animal, and environmental health from global to local levels (1, 6–8). Growing concerns, including emerging infectious diseases, antimicrobial resistance, climate−driven ecological shifts, and zoonotic spillovers, underscore the need for professionals trained to operate across disciplinary boundaries and contribute to Sustainable Development Goals (1, 7, 9). For this perspective, we not only share our experience with the development and execution of One Health IPE activities in several key areas but also propose strategies for how professional students can implement One Health in practice.

## Accreditation

Numerous professional accrediting organizations mandate IPE, with the ultimate goal of improving the quality of patient care, patient safety, and overall health outcomes. Programs that focus on human healthcare, including nursing, medicine, and pharmacy, have requirements for IPE; however, many of the respective accrediting bodies may not specify what fulfills their requirements (Table 1).

**TABLE 1:** Lists the range of students per cohort by professional degree.

Program	Range of students per cohort	Accreditation requires IPE	IPE wording	Standard #	Source/ reference
Allopathic medicine (MD)	50-365	Y	Students must learn to function collaboratively on health care teams with other health professionals	Standard 7.9	AAMC, LCME
Dental hygiene	N/A	Y	Graduates must be competent in interprofessional communication and collaboration.	Standard 2-15	CODA
Dentistry	35-372	Y	Graduates must be competent in communicating and collaborating with other members of the health care team.	Standard 2-20	CODA
Dietitian/nutrition	N/A	Y	Students must identify, describe, and engage in interprofessional team functions.	KRDN 2.5; CRDN 2.4	ACEND
Medical laboratory science	N/A	Y	Requires collaboration in diagnosis/treatment and consultative interactions within healthcare teams.	Standard II	NAACLS
Nurse practitioner	N/A	Y	Curriculum includes planned experiences fostering interprofessional collaborative practice.	Standard III-J	CCNE, ACEN
Nursing	N/A	Y	Curriculum includes planned experiences fostering interprofessional collaborative practice.	Standard III-J	CCNE, ACEN
Occupational therapy (doctorate)	5-144	Y	Demonstrate knowledge of the principles of intraprofessional and interprofessional team dynamics to perform effectively in different team roles to plan, deliver, and evaluate patient- and population-centered care as well as population health programs and policies that are safe, timely, efficient, effective, and equitable.	Standard B.3.22	ACOTE
Occupational therapy (masters)	12-190	Y	Demonstrate knowledge of the principles of intraprofessional and interprofessional team dynamics to perform effectively in different team roles to plan, deliver, and evaluate patient- and population-centered care as well as population health programs and policies that are safe, timely, efficient, effective, and equitable.	Standard B.3.22	ACOTE
Optometry	28-127	Y	By the time of graduation, students must be able to apply knowledge of interprofessional collaborative care, ethics, and medico-legal aspects for the delivery of optometric care.	Standard 2.18	ASCO, ACOE
Osteopathic medicine	81-271	Y	Curriculum must include interprofessional education preparing students to collaborate on health care teams.	Standard 6.4	AACOM, AOA
Pharmacy	7-249	Y	Curricula must prepare students for team-based, interprofessional collaborative practice.	Standard 11	PharmCAS, ACPE
Physical therapy	5-104	Y	The didactic and clinical education curriculum includes intra-professional (PT/PTA) and interprofessional (PT with other professions/ disciplines) learning activities that are based on best-practice and directed toward the development of intra-professional and interprofessional competencies including, but not limited to, values/ethics, communication, professional roles and responsibilities, and teamwork.	Standard 6F	CAPTE
Physician assistant	13-169	Y	Curriculum prepares students to work collaboratively in interprofessional patient-centered teams.	B2.10	PAEA, ARC-PA
Podiatry	40-125	Y	Demonstrate communication and interpersonal skills that result in relevant information exchanges and decision-making with patients, their families, and members of the healthcare team.	Domain IV	AACPM
Veterinary medicine	75-225	N	Instruction in the contribution of the veterinarian to the overall public and professional healthcare teams	Standard 9	AAVMC, AVMA

N/A indicates that information about cohort sizes for the program could not be found. The table also includes information regarding Interprofessional Education (IPE) along with how the program’s accreditation standard is worded. The last column contains the abbreviation for the accrediting body source/reference for cohort size and IPE requirements.

Other components of One Health, namely animal and environmental health, do not have similar IPE requirements. Currently, the American Veterinary Medical Association Council on Education does not stipulate IPE as a compulsory element of the veterinary curriculum, but it does require instruction addressing the contribution of the veterinarians to the overall public and professional healthcare teams (Table 1). A survey of veterinary schools indicated that only 51% of programs offered IPE (1). Contrary to professional programs for human and animal health, there is no terminal professional degree for environmental health, and training in this field encompasses a variety of disciplines, including biology and engineering, which can be obtained through a baccalaureate degree program. Students in environmental health fields may choose to pursue additional education through a graduate degree program (e.g., MS, Ph.D.) which focus heavily on research as opposed to professional degrees which incorporate clinical training.

## Attendance

There are arguments both for and against mandatory attendance in clinical education, but we feel that attendance should be mandatory for One Health IPE activities (10, 11). The enforcement of mandatory attendance presents challenges, as students frequently express dissatisfaction with compulsory participation. The biggest logistical challenge the authors encountered with mandatory attendance stemmed from how to record and enforce attendance when hundreds of students from different programs participated in IPE activities (Table 1). QR codes in slides are useful as most students can scan the codes with their phones, but there is also the potential for students to share the QR code with peers who are not present. Similarly, many university buildings employ badge scanning, which might produce a log, but this can become problematic when students try to scan in simultaneously prior to the start of the activity. Although many virtual meeting platforms can track participants, they present challenges in other areas. One alternative strategy could involve students maintaining a log of their extracurricular engagements.

Students may perceive mandatory participation as burdensome with some individuals adversely impacted by attendance hurdles (10, 11). We noted that students were particularly troubled when expectations varied among the different programs regarding participation. These discrepancies can create resentment and undermine interprofessional relationships. It is crucial to cultivate student buy-in regarding the necessity of mandatory attendance, which may require increased faculty effort and school administration backing. The development of a singular IPE course for all students can also mitigate these issues by creating clear, uniform expectations for all students that participate in IPE.

Students generally favor smaller group activities over large lecture-based classes, but issues can arise from the lack of resources such as rooms and facilitators to support this preference (12, 13). Organizing small group meetings and course work outside of the classroom environment is complicated by jam-packed student schedules. One solution would be the establishment of set times when resources are needed and/or students could meet with their small groups.

## Types of activities

Multiple educational strategies such as lectures, small group work, practice-based learning, and collaborative exercises foster active participation and skill development among different professions (2, 3, 14, 15). Additionally, community-based service-learning experiences have been employed by several programs to provide diverse, immersive interdisciplinary opportunities for learners to engage with the social and environmental determinants of health (5, 16–19).

We have successfully employed a variety of methods, from guest lecturers to gamification, to engage students during IPE. Guest lecturers can play a crucial role in exemplifying One Health interprofessional engagement in real-world contexts. Lectures also provide the benefit of being logistically simple, but student participation may be limited in this format. Lecturers should be encouraged to use polling or clickers to increase student participation and create a more dynamic learning environment.

Small groups are instrumental in promoting cross-disciplinary engagement, which explains their widespread application in IPE settings (2, 4, 5, 13). Based upon the authors’ experience, the ideal group size for effective discussion and collaboration is typically between six and eight participants. However, organizing such groups can be challenging, particularly when considering the total number of students. As cohort sizes vary across different professional programs and disciplines, achieving equal representation in each group is nearly unattainable (Table 1). The inclusion of students at different levels of education (e.g., baccalaureate and professional students) have also negatively impacted group dynamics. These disparities may unintentionally lead to feelings of intimidation during discussions. Early introduction of IPE into the curriculum may be useful in reducing negative attitudes and avoiding stereotypes (4).

Additional issues that may complicate in-person discussion-based small group activities include: (1) finding enough facilitators; (2) having facilitators who can effectively guide discussions; and (3) having enough physical space to accommodate the number of small groups (12). The authors used large classrooms to accommodate multiple small groups, but conversations tended to bleed across various groups.

Gamification has been demonstrated to be an effective approach for fostering group cohesion and enhancing comfort levels; however, it should not be implemented without prior teamwork experience. Englar et al. suggest that IPE should initially focus on clarifying the distinct roles of various professions before engaging in more intense activities (20). In a One Health IPE activity developed by authors that had 51 groups meeting and collaborating for the first time on an escape room activity, none of the groups were able to crack to code within the specified time.

Students often indicate a preference for virtual courses, which overcome some of the barriers encountered with in-person activities. Team members frequently exchange information and collaborate on presentations and other assignments. However, this can sometimes lead to a divide-and-conquer approach within groups, which defeats the purpose of IPE. Students may also feel pressured to report participation from all team members, even when some students are not actively involved.

## Faculty and institution commitments

To be truly considered a “One Health” IPE activity for students, faculty members with expertise in human, animal, and environmental health should be recruited to create equity among the three pillars of One Health (7). Universities focusing on clinical education may lack expertise in animal and environmental health because of the absence of such educational programs within their institutions (12, 21). Currently, only 37 colleges or schools of veterinary medicine in the United States are accredited by or have gained “reasonable assurance” from the American Association of Veterinary Medical Colleges (22). Universities focused on clinical education may lack undergraduate programs with an environmental health connection, potentially leading to the environment being only defined in the context of human health (23). Professional programs should evaluate whether activities are truly “One Health” in nature and creatively address any deficiencies.

Other programs face challenges within the curriculum itself. A survey of 133 American medical schools found that only 56% included One Health in some form, ranging from minor references in microbiology to elective courses in One Health while accredited veterinary schools are required to incorporate public health and teach students about the interrelationship between animals and the environment (21). Professional programs often have deeply ingrained structures and requirements that make it challenging to integrate new interdisciplinary topics, such as those encompassing social, environmental, and biological components of health. Resistance to the integration of a systems-based approach, which is crucial for addressing complex environmental health issues, further complicates this integration (24, 25).

Faculty workload can reach unsustainable levels, depending on the number of participants and the type of activity. For example, designing and executing an activity, such as an escape room, can take months. Once developed, it needs to be pre-tested by a diverse group of students, or even staff and faculty to ensure that it functions as intended. Grading assignments is another facet that can create significant challenges for faculty members. One of the authors devised a rubric with comprehensive responses to facilitate the efficient and consistent grading of 100 small group PowerPoint presentations by faculty of varying backgrounds for a One Health conference. Another author grades almost 4,300 written assignments per IPE course singlehandedly, which equates to over 210 h devoted to grading if spending only an average of three minutes per assignment.

Determining the optimal number and composition of faculty for the implementation of IPE presents significant challenges. Involving an excessive number of faculty members may result in complications such as prolonged timelines and conflicting priorities. Conversely, assigning the responsibility for IPE activities to a single faculty member is disadvantageous for several reasons. First, it is improbable that a single faculty member possesses comprehensive expertise in all aspects of One Health. Second, managing a large course may lead to an uneven distribution of workload and potential faculty burnout; therefore, it is advisable to appoint a co-course director (26).

Although some accrediting bodies require IPE, educational institutions may not provide sufficient support for the course. There is often a lack of an instructional model that effectively incorporates IPE, resulting in persistent silos and minimal interprofessional collaboration among faculty and departments. Lack of support can also lead to the belief that IPE is an “add-on” as opposed to being integrated within the curriculum (1). Owing to competing priorities within programs and institutions, funding for the course may not exist or may be inadequate compared with the number of students involved (13).

## Student feedback

Gathering student feedback is essential for enhancing the implementation of One Health IPE; however, course evaluations may not adequately capture critical information related to One Health knowledge, as they frequently focus on course pedagogy (4, 12). Extracurricular events and conferences must be planned at least several months in advance to obtain the necessary approval from an institutional review board to survey students.

**FIGURE 1:**
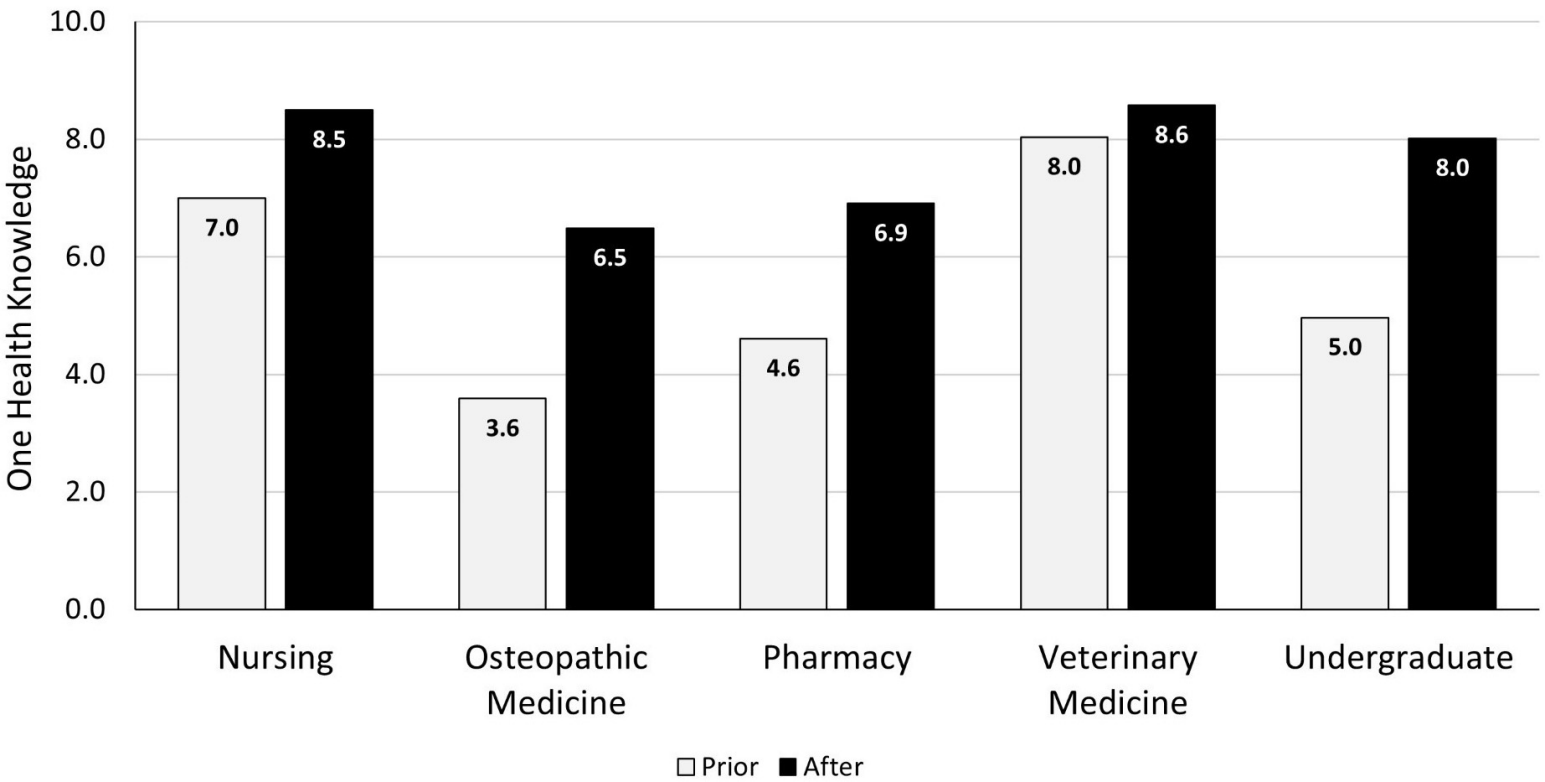
2018 pre-and post-conference One Health knowledge mean scores by academic program.

One of our institutions developed a survey to obtain student feedback for two conferences held in 2018 and 2019 that focused on One Health IPE. Participants were queried regarding their knowledge of One Health before and after the conferences. In 2018, the conference included a small group case competition and seven discussion-based scenarios. Students assessed their pre-conference One Health knowledge with a mean score of 5.03 (range 1–10) and post-conference knowledge with a mean score of 7.42 (range 1–10), with results varying among the different professional groups attending the conference (Figure 1). In 2019, the conference format had students rotating between lectures and a small group “escape room-type” activity. Students rated their pre-conference One Health knowledge with a mean score of 6.69 (range 1–10) and their post-conference knowledge with a mean score of 8.19 (range 1–10). Part of this increase can likely be attributed to students attending consecutive events; however, only aggregate data is currently available, which hinders the ability to determine how scores were impacted by students attending both years. Seventy-six percent of the students attending the 2019 event also expressed a preference for the game format and believed that input from their area of expertise was crucial for solving the case.

Students have also noted that small group interdisciplinary interactions are almost invariably beneficial, gaining substantial knowledge about other professions that they previously had a limited understanding of (3, 12, 14, 20, 27). While students value these interactions, feedback has also identified challenges, including unclear instructions, uneven preparation, schedule conflicts, and perceptions that IPE is less important than discipline-specific coursework.

Professional students have packed schedules within their primary program, having to juggle classes, hands-on skill development, and exams to progress to the next step of their program. Focusing on individual exam success can affect students’ preparation for and engagement in collaborative group assignments. Our experience has also taught us that students will be dismissive of lectures, courses, and anything else that might be perceived as superfluous to their clinical training; therefore, it is imperative to demonstrate the importance of One Health to them as future practitioners.

## Additional comments

We have also experienced challenges associated with Wi-Fi bandwidth when students are required to log in or submit responses online, as well as misunderstandings of terminology across various professions. Moreover, several issues have been noted that underscore discrepancies between expectations when planning and actual experiences: not all students engage in preparatory reading, students often focus on their own discipline and the medical aspects of activities, there is a lack of comprehension of certain concepts, materials may be discarded during room resets, and there is insufficient communication among the team members.

## Discussion

Both IPE and the One Health approach emphasize collaboration, teamwork, and critical thinking to address complex, multifaceted issues, with IPE focusing more on patient-centered care and One Health being framed in terms of population or global health to optimize human, animal, and ecosystem wellbeing to tackle “wicked problems” (1, 8, 9, 14). Although One Health learning experiences are essential for enhancing knowledge across various professions, their implementation can be challenging without structural, cultural, and pedagogical alignment. Challenges such as scheduling conflicts, uneven institutional expectations, varying levels of faculty expertise and interest, differences in student training levels, and large cohort sizes have been documented by the authors and others (1, 6, 12, 15). Despite these challenges, we posit that a viable path exists for the successful adoption of One Health IPE within academic settings.

First, institutional support is crucial (1, 12, 13, 28). Administrative infrastructure must ensure protected time for faculty, align funding with the number and type of students enrolled in IPE, mandate program participation if necessary for accreditation purposes, provide coordination across colleges/programs, and develop consistent policies to signal that One Health IPE is an organizational priority (1, 6). Without such support, implementation relies on individual advocates and is unsustainable. Faculty modeling is also pivotal for the effective implementation of One Health IPE. Students are more likely to adopt behaviors as professional norms when instructors engage in genuine interdisciplinary collaboration, share expertise, acknowledge uncertainty, and treat peers as equals, (12, 29, 30). Most professional students will not practice at a global or population level; thus, they prioritize individual patient care, which should be considered when encouraging students to embrace One Health (1, 6).

One Health integration into clinical practice enhances healthcare by fostering intersectoral collaboration that emphasizes the interconnectedness of humans, animals, and the environment. General practitioners and veterinarians, as frontline healthcare providers, play complementary roles in diagnosing and managing zoonotic diseases, with veterinarians often demonstrating greater expertise and confidence in zoonoses identification and prevention (19, 31). However, effective cross-professional collaboration remains limited, partly due to gaps in understanding each other’s roles and professional humility, which are critical for improved clinical outcomes (19). One Health approaches also expand diagnostic capabilities by encouraging clinicians to consider environmental and animal factors contributing to disease, thereby promoting holistic patient care (32, 33). Sixty-eight percent of people in North America live with at least one pet so human healthcare should incorporate asking about pets in the household (34). Asking about pets improves recognition and treatment of zoonotic diseases, provide behavioral changes and motivation, act of agents of harm reduction, act as sentinels of disease, and increase patient engagement in a perceived threatening or judgmental environment (18, 31, 34). Additionally, healthcare practices are negatively affecting the environment which ultimately exacerbates human health issues. The healthcare industry is a source of air, soil, and water pollution and the built-hospital environment can also impact human health (33, 35–39). Implementing green healthcare practices and providing education about environmental health effects should be considered integral to addressing One Health challenges (32, 37, 38).

This perspective, alongside much of the extant literature on IPE and the One Health approach, has predominantly concentrated on the development and implementation of soft skills such as teamwork. We did include some data about student One Health knowledge; however, there is still a paucity of research examining the comprehension of One Health across various professional domains and the potential future application of One Health principles by students. We propose that subsequent research should explore how the institutional implementation of IPE and One Health affects student attitudes, the enduring integration of One Health concepts into clinical practice, and the extent of familiarity with One Health among practitioners outside academia.

## Data Availability

The data analyzed in this study is subject to the following licenses/restrictions: the data was originally collected in 2018 and 2019. Only aggregate data is available at this time. Requests to access these datasets should be directed to Karen Gruszynski, kgrusz@midwestern.edu.
